# SIRT5 Inhibition Induces Brown Fat-Like Phenotype in 3T3-L1 Preadipocytes

**DOI:** 10.3390/cells10051126

**Published:** 2021-05-07

**Authors:** Francesca Molinari, Alessandra Feraco, Simone Mirabilii, Serena Saladini, Luigi Sansone, Enza Vernucci, Giada Tomaselli, Vincenzo Marzolla, Dante Rotili, Matteo A. Russo, Maria Rosaria Ricciardi, Agostino Tafuri, Antonello Mai, Massimiliano Caprio, Marco Tafani, Andrea Armani

**Affiliations:** 1 Department of Experimental Medicine, Sapienza University of Rome, 00161 Rome, Italy; francesca.molinari@sanraffaele.it (F.M.); serena.saladini@live.it (S.S.); marco.tafani@uniroma1.it (M.T.); 2 Laboratory of Cardiovascular Endocrinology, IRCCS San Raffaele Pisana, 00163 Rome, Italy; alessandra.feraco@sanraffaele.it (A.F.); vincenzo.marzolla@sanraffaele.it (V.M.); massimiliano.caprio@sanraffaele.it (M.C.); 3 Department of Clinical and Molecular Medicine, Sapienza University of Rome, 00189 Rome, Italy; simone.mirabilii@uniroma1.it (S.M.); mariarosaria.ricciardi@uniroma1.it (M.R.R.); agostino.tafuri@uniroma1.it (A.T.); 4 Department of Cellular and Molecular Pathology, IRCCS San Raffaele, 00166 Rome, Italy; luigi.sansone@sanraffaele.it (L.S.); giada.tomaselli@virgilio.it (G.T.); matteo.russo@sanraffaele.it (M.A.R.); 5 Department of Cardiovascular, Nephrologic, Anesthesiologic and Geriatric Sciences, Sapienza University of Rome, 00161 Rome, Italy; enza.vernucci@gmail.com; 6 Department of Chemistry and Technology of Drugs, Sapienza University, P.le Aldo Moro 5, 00185 Rome, Italy; dante.rotili@uniroma1.it (D.R.); antonello.mai@uniroma1.it (A.M.); 7 MEBIC Consortium, San Raffaele Rome Open University, 00166 Rome, Italy; 8 Hematology, “Sant’ Andrea” University Hospital, Sapienza University of Rome, 00189 Rome, Italy; 9 Department of Human Sciences and Promotion of the Quality of Life, San Raffaele Roma Open University, 00166 Rome, Italy

**Keywords:** obesity, adipogenesis, mitochondrial function, adipose tissue, sirtuins

## Abstract

Brown adipose tissue (BAT) activity plays a key role in regulating systemic energy. The activation of BAT results in increased energy expenditure, making this tissue an attractive pharmacological target for therapies against obesity and type 2 diabetes. Sirtuin 5 (SIRT5) affects BAT function by regulating adipogenic transcription factor expression and mitochondrial respiration. We analyzed the expression of SIRT5 in the different adipose depots of mice. We treated 3T3-L1 preadipocytes and mouse primary preadipocyte cultures with the SIRT5 inhibitor MC3482 and investigated the effects of this compound on adipose differentiation and function. The administration of MC3482 during the early stages of differentiation promoted the expression of brown adipocyte and mitochondrial biogenesis markers. Upon treatment with MC3482, 3T3-L1 adipocytes showed an increased activation of the AMP-activated protein kinase (AMPK), which is known to stimulate brown adipocyte differentiation. This effect was paralleled by an increase in autophagic/mitophagic flux and a reduction in lipid droplet size, mediated by a higher lipolytic rate. Of note, MC3482 increased the expression and the activity of adipose triglyceride lipase, without modulating hormone-sensitive lipase. Our findings reveal that SIRT5 inhibition stimulates brown adipogenesis in vitro, supporting this approach as a strategy to stimulate BAT and counteract obesity.

## 1. Introduction

Adipose tissue can be mainly categorized into two distinct types: white and brown adipose tissue (WAT and BAT, respectively) showing different cell morphology and function [1]. WAT is primarily composed of white adipocytes, containing one large cytoplasmic droplet of white-yellow fat, specialized in energy storage and release, whereas BAT consists mainly of brown adipocytes containing multilocular lipid droplets and numerous mitochondria [1]. In the brown adipocyte, mitochondria are deputed to dissipate energy via adaptive non-shivering thermogenesis, due to the presence of the uncoupling protein-1 (UCP-1) in these organelles [2]. Notably, some depots of WAT contain adipose cells termed “beige” or “brite” adipocytes, displaying morphology and function similar to brown adipocytes. Emergence of beige adipocytes in WAT, a process named “browning”, is induced by physiological stimuli, such as cold exposure, diet and physical exercise [3]. A number of drug agents, metabolites and molecular pathways have been shown to regulate browning and lead to the increased thermogenic capacity of adipose tissue [3,4,5,6,7]. Both brown and beige adipocytes play a relevant role in the regulation of whole-body energy homeostasis. In fact, BAT activation and/or the emergence of beige adipocytes in mouse WAT result in energy expenditure enhancement leading to the dissipation of extra-calories, with subsequent reduction of excessive white fat mass and body weight [2]. The discovery of BAT in adult humans, more than ten years ago [8,9], has excited considerable interest in developing pharmacological approaches aimed at stimulating BAT function as novel strategies to treat obesity and related diseases [10].

Sirtuins are a class of NAD^+^-dependent protein deacetylases whose functions strongly affect the metabolism of different tissues, including WAT and BAT [11]. Mitochondrial sirtuins SIRT3 and SIRT5 regulate mitochondrial function and BAT thermogenesis [12,13,14]. In particular, SIRT5 has been shown to regulate the activity of UCP-1, and lack of SIRT5 leads to hypersuccinylation and subsequent impairment in UCP-1 activity [14]. In the BAT of SIRT5 KO mice, the reduced function of UCP-1 and other mitochondrial proteins, due to elevated protein succinylation, results in defective mitophagy, altered mitochondrial respiration and impaired cold adaptation [14]. The clearance of damaged mitochondria through mitophagy contributes to mitochondrial quality, that is a critical requirement for brown adipocyte function [15], and activation of the AMP-activated protein kinase (AMPK), a cellular energy sensor, promotes mitophagy to preserve BAT mitochondrial quality [15]. A number of studies indicate that AMPK is required for brown/beige adipose tissue development and thermogenesis [16]. SIRT5 deficiency has been shown to reduce mitochondrial ATP production, leading to an increase in AMP/ATP ratio and activation of AMPK [17], and suggesting that changes in SIRT5 activity might affect AMPK function and, in turn, mitochondrial activity and the development of brown/beige adipocytes. As observed by Shuai et al., SIRT5 activity is also able to modulate brown adipogenic gene expression by generating histone modifications [18]. A SIRT5 deficiency in mice leads to reduced intracellular concentrations of α-ketoglutarate, resulting in increased methylation levels of histones at the promoters of *Pparγ* and *Prdm16* genes, with subsequent repression of these two drivers of brown adipogenesis [19]. In cancer cell lines and C2C12 myoblasts, treatment with the SIRT5 inhibitor MC3482 has been shown to promote autophagy and mitophagy, through increased ammonia production [20]. Considering the key role played by the autophagic and the mitophagic flux in adipocyte differentiation [21,22] and mitochondrial homeostasis [23,24], pharmacological inhibition of SIRT5 might affect adipogenesis and adipocyte mitochondrial function.

Here we have explored the in vitro effects of the SIRT5 inhibitor MC3482 on brown adipocyte differentiation and functions. We observed that this compound was able to induce a brown fat-like phenotype in 3T3-L1 adipocytes differentiated in vitro, suggesting that treatment with MC3482 may favor brown adipogenesis and represent a valid pharmacological approach against obesity and obesity-related diseases.

## 2. Materials and Methods

### 2.1. Cell Cultures

Mouse 3T3-L1 preadipocytes were purchased from ATCC (American Type Culture Collection) (LGC Standards, Milan, Italy) and cultured according to the manufacturer’s instructions. Briefly, cells were grown until confluence at 37 °C in a 5% CO2 incubator in DMEM (DMEM Glutamax, GIBCO) containing 4.5 g/liter D-glucose, 10% fetal bovine serum (FBS) 100 U/mL penicillin, and 100 g/mL streptomycin (EuroClone, Milan, Italy). After confluence, 3T3-L1 preadipocytes were induced to differentiate by the addition of a cocktail containing 100 µM 3-isobutyl-1-methylxanthine (IBMX), 175 nM insulin, and 250 nM dexamethasone, for 48 h, then incubated for an additional 5 or 8 days in DMEM supplemented with 10% FBS and 175 nM insulin, replaced every 48 h. Since the first 2 days of differentiation are crucial for driving the transcriptional cascade that leads to adipocyte differentiation [25,26], the SIRT5 inhibitor MC3482 (50 μM) was added from the confluence until day 2. During the first 48 h of differentiation, cells were treated with MC3482 every 12 h. Concentration of 50 μmol/liter for MC3482 was already shown as the most effective in inhibiting SIRT5 in a previous study which also reported synthesis and characterization of the compound [20]. The 5-Iodotubercidin (Sigma Aldrich, Milan, Italy) was dissolved in DMSO and added to culture medium at day 0, at a concentration of 0.2 μM, until day 2 of adipocyte differentiation. Primary cultures of murine preadipocytes were prepared from the vascular stromal fraction (SVF) of interscapular (BAT) or inguinal fat depots (iWAT) of 10-week-old male C57BL/6J mice as previously described [27]. C57BL/6J mice were purchased from Charles River Laboratories, (Calco, LC, Italy). Animal procedures were approved by the Italian National Institute of Health Care and Use Committees. As described in another study [27], the proliferation medium of primary adipocyte cultures consisted of DMEM/Ham’s F12 (Invitrogen, Milan, Italy) supplemented with 10% FBS, 100 U/mL penicillin, and 100 μg/mL streptomycin. Adipogenic differentiation was induced by incubating the cells in the same medium supplemented with 0.5 mM IBMX, 125 nM indomethacin, 5 μM dexamethasone, 850 nM insulin, 1 nM triiodothyronine (T3). After 2 days, cells were incubated with DMEM/Ham’s F12 containing 10% FBS, 850 nM insulin, 1 nM T3. Cells were allowed to differentiate until day 6. Differentiating cells were treated with MC3482 during the first 2 days of differentiation.

### 2.2. Gene Expression Analysis

Total RNA was isolated from mouse primary adipocyte cultures by Trizol (Life Technologies, Milan, Italy) according to the manufacturer’s instructions and analyzed by RT-qPCR. The yield of RNA was analyzed by using NanoDrop 1000 Spectrophotometer (Thermo Scientific, Milan, Italy). Total RNA (1 μg) was treated with DNase 1 (Invitrogen, Milan, Italy) and reverse transcribed using GoScript Reverse Transcription System (Promega, Milan, Italy) according to the manufacturer’s instructions. qPCR was performed by using the Mx3000P LightCycler instrument (Stratagene, Milan, Italy) with GoTaq qPCR Master Mix (Promega). The thermocycle conditions were 95 °C for 3 min, followed by 40 cycles of 95 °C for 5 s and 60 °C for 20 s, then followed by 95 °C for 1 min, 55 °C for 30 s, 95 °C for 30 s. Sequences of the primers are given in Table S1. Expression of each target gene was normalized to the TATA-box binding protein (TBP) transcript.

### 2.3. Immunoblot Analysis

Cells and specimens of mouse fat depots were lysed in HNTG Lysis Buffer (50 mM HEPES, pH 7.4, 150 mM NaCl, 10% Glycerol, 1% Triton) containing Protease Inhibitor Cocktail (Sigma-Aldrich, Milan, Italy) and Phosphatase Inhibitor Cocktail (Sigma-Aldrich, Milan, Italy). A clear supernatant surrounded by a fat cake on the top, was obtained by centrifugation of lysates at 14,000× *g* for 20 min at 4 °C. Fat cake was removed and the supernatant was transferred into a clean tube. Protein concentration in the supernatant was determined by BCA Bradford protein assay (Bio-Rad, Hercules, CA, USA). Total lysates were run on pre-cast gradient gels (4–20%-BioRad). Proteins were transferred to nitrocellulose membranes (Bio Rad Nitrocellulose Membrane, 0.45 µm) and were blocked overnight at 4 °C with 5% non-fat milk or 5% BSA in TTBS (TBS with 0.05% Tween 20-UCS Diagnostics). Incubation with primary specific antibodies was performed overnight at 4 °C and then horseradish peroxidase conjugated secondary antibodies was performed in blocking solution for 1 h at room temperature. Equal loading of samples was confirmed by actin normalization. We used antibodies against LC3B (NB 600-1384; Novus Biologicals, Abingdon, UK), UCP1 (ab10983; Abcam, Cambridge, UK), A-FABP (B-4) (sc-271529; Santa Cruz Biotechnology, Heidelberg, DE), C/EBP-α(D-5) (sc-365318; Santa Cruz Biotechnology, Heidelberg, DE), PGC-1α (ab3242; Millipore, Milan, Italy), TFAM (sc-23588; Santa Cruz Biotechnology, Heidelberg, DE), ATGL (30A4) (2439; Cell Signaling Technology, Denver, CO, USA), pATGL (ser406) (ab135093; Abcam), pHSL660 (4126T; Cell Signaling Technology), HSL (4107; Cell Signaling Technology), PPAR-γ (E-8) (sc-7273; Santa Cruz Biotechnology), SIRT5 (ab13697; Abcam), β-actin (A-5316; Sigma-Aldrich), pAMPKα(Thr172) (2535; Cell Signaling Technology), AMPKα (2603; Cell Signaling Technology), pULK1(Ser555) (5869; Cell Signaling Technology), ULK1 (8054; Cell Signaling Technology), BNIP3 (sc-56167; Santa Cruz Biotechnology), α-tubulin (T5168; Sigma-Aldrich), p-ACC1 (3661; Cell Signaling Technology). Immunoreactive bands were visualized by Luminata Forte Western HRP Substrate (Millipore) and Clarity Western ECL Substrate (BioRad, Milan, Italy). Gel image acquisition and densitometric scanning analysis were performed by using ImageQuant TL (GE Healthcare, Chicago, IL, USA).

### 2.4. Mitochondrial DNA Content Analysis

Mitochondrial and nuclear DNA of 3T3-L1 cells were isolated by using the DNeasy DNA isolation kit (Qiagen, Hilden, Germany) and were analyzed by qPCR using specific primers for the mitochondrial DNA region D-loop and for the nuclear gene β-2-microglobulin (β2M). The sequences of the primers are given in Table S1. Reaction parameters were those mentioned above (in “gene expression analysis” paragraph). Mitochondrial DNA content is expressed as the mitochondrial D-loop levels normalized to nuclear β2M levels.

### 2.5. Immunofluorescence and Combined Oil Red O Staining

The 3T3-L1 preadipocytes were seeded in 35 mm glass bottom dishes and cultured as described above. At day 10 of differentiation, cells were fixed with 3.7% formaldehyde in PBS for 20 min at room temperature and then washed four times with PBS. Then, cells were treated with 0.2% Triton X-100 in PBS for 7 min and washed with three exchanges of PBS for 5 min. Fixed cells were incubated for 1 h at RT in 1% BSA to block nonspecific antibody binding and then were incubated with primary antibody in 1% BSA overnight at 4 °C (anti-ATGL, 1:100 dilution, Cell Signaling Technology; anti-UCP1, 1:200 dilution, Abcam; anti-TFAM, 1:100 dilution, Santa Cruz Biotechnology). After overnight incubation with antibodies, cells were washed in PBS, incubated for 1 h at RT with labeled anti-goat (Alexa Fluor 488; Molecular Probes, Eugene, OR, USA) or anti-rabbit (FITC 711-035-152; Jackson ImmunoResearch Laboratories, West Grove, PA, USA) secondary antibodies. To permit examination of lipids stained by Oil Red O together with ATGL protein, after 15 min washing with three exchanges of PBS, glass bottom dishes were filled with the working solution of Oil Red O for 10 min and were rinsed twice with deionized water, followed by 10 min with running tap water (Oil Red O Stock: Sigma O-0625). Subsequently, cells were stained for 3 min with DAPI to label nuclei. The samples were mounted in Aquamount (Sigma F4680). For the standard protocol of immunofluorescence, Oil Red O staining step was skipped. For quantitative evaluation of Oil Red O staining, every image was taken on the Nikon Eclipse Ti2 and analyzed with the software ImageJ 1.37v (National Institutes of Health, Bethesda, MD, USA) as previously described [28].

### 2.6. Transmission Electron Microscopy Analysis

Terminally differentiated 3T3-L1 cells were prepared as described by Polletta et al. [20]. Briefly the cells were pelleted after trypsinization, fixed overnight at 4 °C in 2% glutaraldehyde with 1% tannic acid in 0.1 M sodium cacodylate, pH 7.3, rinsed in sodium cacodylate buffer 3 times and incubated for 2 h at room temperature in 2% osmium tetroxide. After 3 washes in distilled water, cells were exposed to 1% uranyl acetate in water for 15 min at room temperature, rinsed again twice in distilled water and spun down into 3% agarose at 45 °C, and cooled to form blocks. The blocks were dehydrated in ascending alcohol concentration, embedded in Spurr’s low viscosity media. After polymerization overnight at 65 °C, were cut on a ultramicrotome (Leica EM UC7; Leica, Wetzlar, Germany) and picked up on copper grids. The grids were then post-stained with uranyl acetate and lead citrate before TEM imaging. The sections were observed in a JEM- 1400 TEM (JEOL Milan, Italy).

### 2.7. Oxygen Consumption Rate (OCR) Analysis

Mitochondrial respiration rates were measured using the Seahorse XF24 Extracellular Flux Analyzer (Agilent Technologies, Santa Clara, CA, USA) as previously published [29]. Briefly, 5 × 10^3^ 3T3-L1 cells/well were seeded and differentiated with or w/o MC3482, directly in the XF24 dedicated plates. Before the assay, cell medium was replaced with unbuffered DMEM medium supplemented with 2 mM L-glutamine, 11 mM Glucose and 1.2 mM Pyruvate adjusted to pH 7.35, and the plates were incubated for 30 min at 37 °C in a CO_2_-free incubator. Oxygen consumption rates (OCR) were measured for the basal state and following the sequential injection of oligomycin (1 μM), carbonyl cyanide-4-(trifluoromethoxy) phenylhydrazone (FCCP) (3 μM), and a mix of Antimycin A (2 μM) and Rotenone (2 μM) (all reagents from Merck KGaA, Darmstadt, Germany) in each well, according to Seahorse Mito Stress Test protocol [30]. Results from the experiments were analyzed through the Seahorse Wave software (Agilent Technologies, Santa Clara, CA, USA).

### 2.8. Lipolysis Assay

The 3T3-L1 preadipocytes were grown and differentiated in 96-well cell culture plates in presence or absence of MC3482 (during the first two days of differentiation). On day 10 of differentiation, cell culture medium was replaced with 150 µL of lipolysis buffer (K577; BioVision, Milpitas, CA, USA). In order to induce the lipolysis, 3T3-L1 were stimulated with isoproterenol (ISO, 100 nM) for 3 h. At the end of ISO stimulation, the amount of glycerol released into the lipolysis buffer was determined using the lipolysis colorimetric assay kit (K577, BioVision), following the manufacturer’s protocol. Thereafter, cells were washed twice with cold PBS and lysed on ice with HTNG cell lysis buffer containing protease and phosphatase inhibitors for the total protein quantification. Glycerol amount was expressed as pmol/µg of total protein.

### 2.9. Statistical Analysis

Data are reported as mean +/− standard error of the mean (SEM). Before using parametric tests, we used the Shapiro−Wilk W-test to verify the assumption of normality The Student paired t test was used to determine any significant differences between untreated and MC3482-treated cells treatment. Values of <0.05 were considered significant. Version of Prism 8.0 (GraphPad, San Diego, CA, USA).

## 3. Results

### 3.1. Protein Expression of SIRT5 in Distinct Fat Depots and Adipocyte Cultures

**Figure 1:**
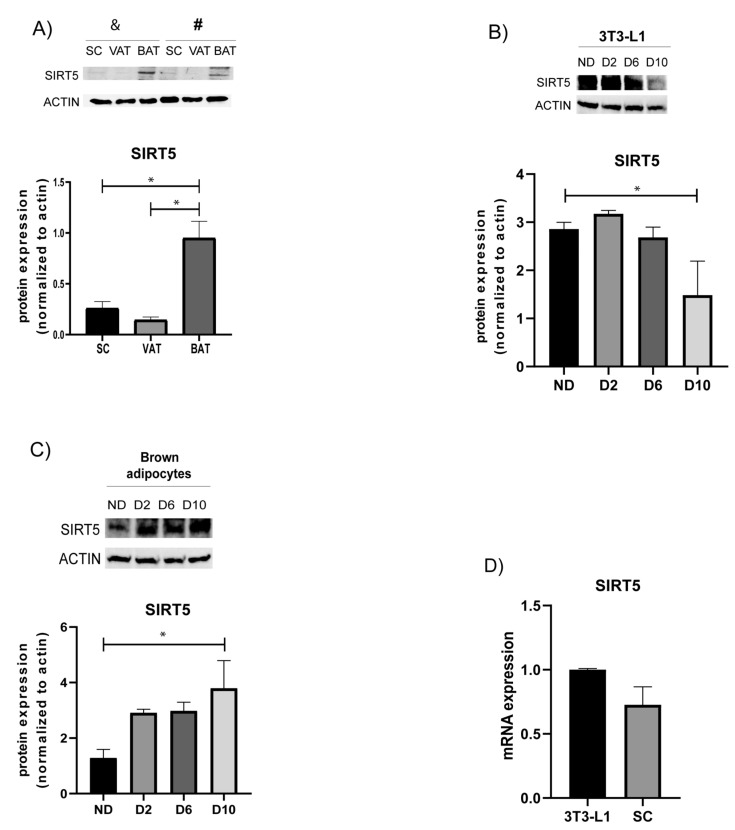
Characterization of SIRT5 expression in mouse fat depots, white and brown adipocytes during in vitro differentiation. (**A**) SIRT5 protein expression in distinct fat depots from male C57BL/6J mice (SC = subcutaneous, (iWAT)); VAT = visceral adipose tissue; BAT = brown adipose tissue). Western blot analysis on two distinct mice (indicated with the symbols & and #) showed higher levels of SIRT5 in BAT compared with those observed in VAT and SC depots. * *p* < 0.05 vs. BAT. (**B**) SIRT5 protein levels in 3T3-L1 cells, nondifferentiated (ND), at day 2, 6, and day 10 of differentiation, were detected by Western blot analysis. * *p* < 0.05 vs. D10. (**C**) SIRT5 protein expression during differentiation of mouse primary preadipocytes derived from BAT of male C57BL/6J mice. * *p* < 0.05 vs. D10. (**D**) Analysis of SIRT5 transcript levels in terminally differentiated 3T3-L1 adipocytes (day 10) and in SC fat depots.

Previous work has shown that SIRT5 is expressed in mouse WAT and BAT [31]. We dissected distinct fat depots from male C57BL/6 mice and we observed higher levels of SIRT5 protein in BAT, compared with those observed in visceral (VAT) and inguinal (subcutaneous, SC) fat depots (Figure 1A), suggesting a relevant role for SIRT5 in BAT function. To confirm the expression of SIRT5 in adipose cells, we analyzed SIRT5 protein expression both in 3T3-L1 adipose cells (Figure 1B) and in mouse primary adipocytes (Figure 1C) derived from BAT of male C57BL/6 mice and induced to undergo terminal differentiation. Both 3T3-L1 and primary brown preadipocytes (lane ND in Figure 1B,C) showed detectable expression of SIRT5 although revealing an opposite expression profile during differentiation. Comparable levels of SIRT5 transcript were detected between terminally differentiated 3T3-L1 adipocytes and SC WAT (Figure 1D). Higher levels of SIRT5 were detected in the early stages during 3T3-L1 preadipocyte differentiation (Figure 1B), as well as in terminally differentiated brown adipocytes (Figure 1C), suggesting that reduced SIRT5 activity at the beginning of differentiation might play a role in brown adipogenesis.

### 3.2. Pharmacological SIRT5 Inhibition Stimulates Brown Adipogenesis and Mitochondrial Activity

**Figure 2:**
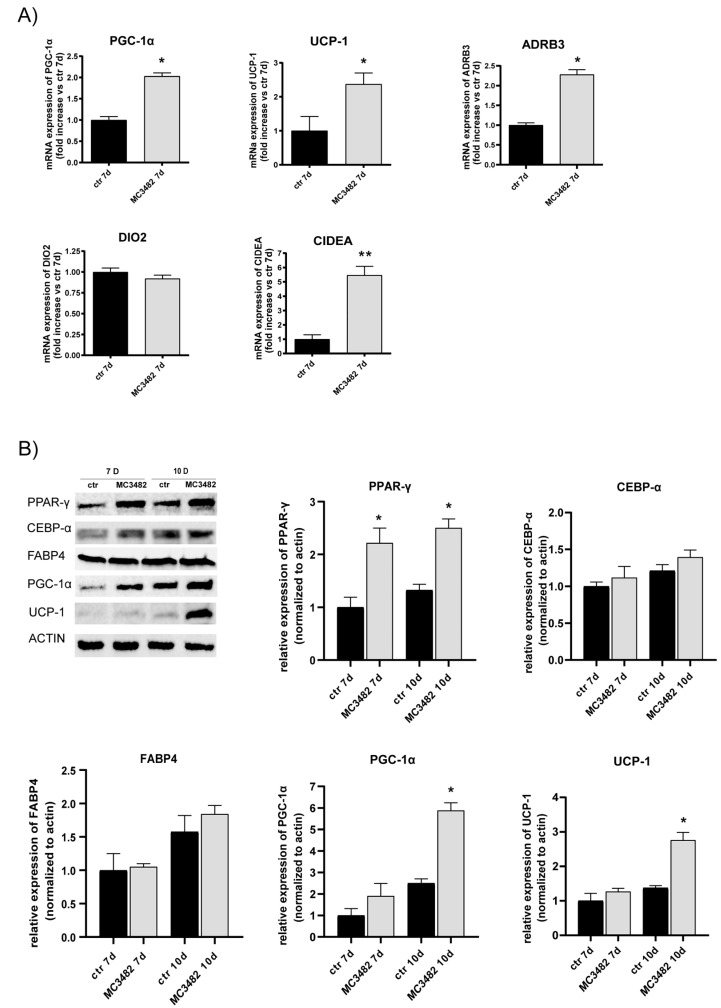
Effects of SIRT5 blockade on adipocyte differentiation. (**A**) PGC-1α, UCP-1, ADRB3, DIO2 and CIDEA mRNA expression levels were evaluated at day 7 of differentiation by RT-qPCR in primary preadipocytes derived from iWAT and induced to differentiate with or without MC3482. (**B**) Western blot analysis of adipogenic marker expression in 3T3-L1 cells induced to differentiate with or without MC3482. Western blot analysis was performed by ImageQuant software. Values are expressed as means ± SEM of three independent experiments. * *p* < 0.05, ** *p* < 0.01 vs. untreated control (ctr) at corresponding time points.

In order to investigate the effects of SIRT5 activity on adipogenesis, we treated cultures of primary preadipocytes, purified from inguinal fat depots of male C57BL/6J mice, with the SIRT5 inhibitor MC3482. Previous work has shown that such mouse primary preadipocytes, derived from inguinal (subcutaneous) white adipose tissue (iWAT), are able to differentiate into brown adipocytes in response to certain stimuli [27,32]. Treatment with MC3482 at the early stage of differentiation led to increased transcript levels of uncoupling protein 1 (UCP-1), peroxisome proliferator-activated receptor-γ coactivator 1-α (PGC-1α), β-3 adrenergic receptor (ADRB3) and cell death-inducing DFFA-like effector A (CIDEA) (Figure 2A), revealing that MC3482 stimulated expression of BAT markers [33].

The effects of SIRT5 inhibition on adipose differentiation were also investigated in the 3T3-L1 mouse preadipocyte cell line induced to differentiate in the presence of MC3482 (Figure 2B). Treatment with MC3482 during the early stages of differentiation (0-2 days of differentiation) led, in terminally differentiated 3T3-L1 adipocytes, to increased levels of PPAR-γ, UCP-1 and PGC-1α proteins, required for brown adipocyte differentiation and thermogenic function [34,35], thus indicating that SIRT5 inhibition promoted 3T3-L1 preadipocyte differentiation into brown-like adipocytes (Figure 2B). Protein levels of adipogenic transcription factors not specifically involved in brown adipocyte differentiation, such as CEBP-α and FABP4 [36,37], were not altered by MC3482 (Figure 2B). Notably, MC3482 treatment of 3T3-L1 preadipocytes resulted in increased transcript levels of two master regulators of brown adipogenesis, PRDM16 and PPAR-γ, at day 7 of differentiation (Figure S1A), confirming that SIRT5 modulation affects expression of key transcription factors of brown fat differentiation [18,38]. As observed in mouse primary adipocytes (Figure 2A), MC3482 treatment in 3T3-L1 adipocytes led to higher transcript levels of browning-related markers, such as PGC-1α, UCP-1, and CIDEA (Figure S1B).

**Figure 3:**
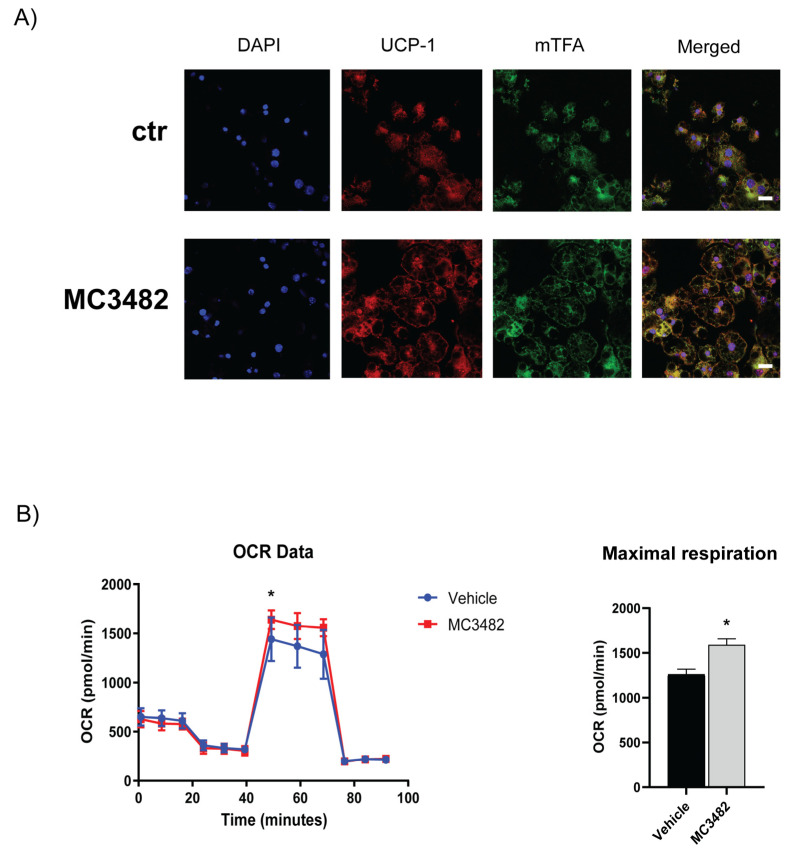
MC3482 treatment enhances brown adipocyte marker expression and stimulates mitochondrial activity. (**A**) Immunofluorescence labelling showing the expression of UCP-1 and mTFA protein levels in terminally differentiated 3T3-L1 adipocytes (day 10) treated with MC3482 compared with control cells (ctr). Nuclei were stained with DAPI (blue). Scale bars = 20 µm. (**B**) Oxygen consumption rates (OCR) analysis performed in 3T3-L1 cells treated with MC3482 vs. vehicle, at day 10 of differentiation. A representative experiment among 3 is shown. Right panel represents maximal respiration data obtained from 3 independent experiments. Values are expressed as means ± SEM. * *p* < 0.05 vs. vehicle.

Immunofluorescence studies confirmed the increased abundance of UCP-1 protein, further indicating the presence of brown-like adipocyte in 3T3-L1 cultures differentiated in the presence of MC3482 (Figure 3A). In addition, we detected a rise in mitochondrial transcription factor A (mTFA) protein levels (Figure 3A) suggesting enhancement in mitochondrial biogenesis [39], as also indicated by the increased levels of PGC-1α transcript and protein, upon treatment with MC3482 both in mouse primary adipocytes and in 3T3-L1 adipocytes (Figure 2). Interestingly, MC3482 treatment also promoted physical interaction between lipid droplets (LDs) and mitochondria (Figure S2A), a phenomenon that occurs during brown adipocyte differentiation [40]. The number of mitochondria bound to LDs increases at the later stages of brown adipocyte differentiation and, as shown by Cui et al., such association may allow fatty acids to move from the LDs to mitochondria and undergo β-oxidation [40]. Such physical interaction was stimulated by MC3482 in terminally differentiated adipocytes, confirming that SIRT5 inhibition promoted brown-like adipocyte features in 3T3-L1 adipocytes. In addition, TEM analyses showed that MC3482 treatment resulted in the appearance of round-shaped mitochondria (Figure S2B) which are associated with increased activation of UCP-1 and conversion of white to brite adipocytes [41,42]. These observed changes in mitochondrial architecture, resulting from increased fragmentation, have been reported to promote mitochondrial uncoupling and energy expenditure [41,42]. Increased levels of Bcl2/adenovirus E1B 19-kDa interacting protein 3 (BNIP3) (Figure S3B), which has been found to favor mitochondrial fragmentation, may contribute to MC3482-driven modifications of the mitochondrial shape (Figure S2B).

Analyses of oxygen consumption rate (OCR) were performed to analyze mitochondrial function. As compared with the vehicle, 3T3-L1 preadipocytes treated with MC3482 showed an increase in maximal respiration stimulated by the mitochondrial uncoupler FCCP (Figure 3B) with a consequent increase in spare respiratory capacity (Figure S3A). No differences induced by MC3482 were observed in the other mitochondrial parameters, such as basal respiration, proton-leak and ATP-coupled respiration (Figure S3A). Overall, these OCR measurements indicated that MC3482 led to more efficient mitochondrial activity, a key feature of brown adipocytes [43].

### 3.3. Pharmacological SIRT5 Inhibition Stimulates Mitophagy and Mitochondrial Biogenesis

**Figure 4:**
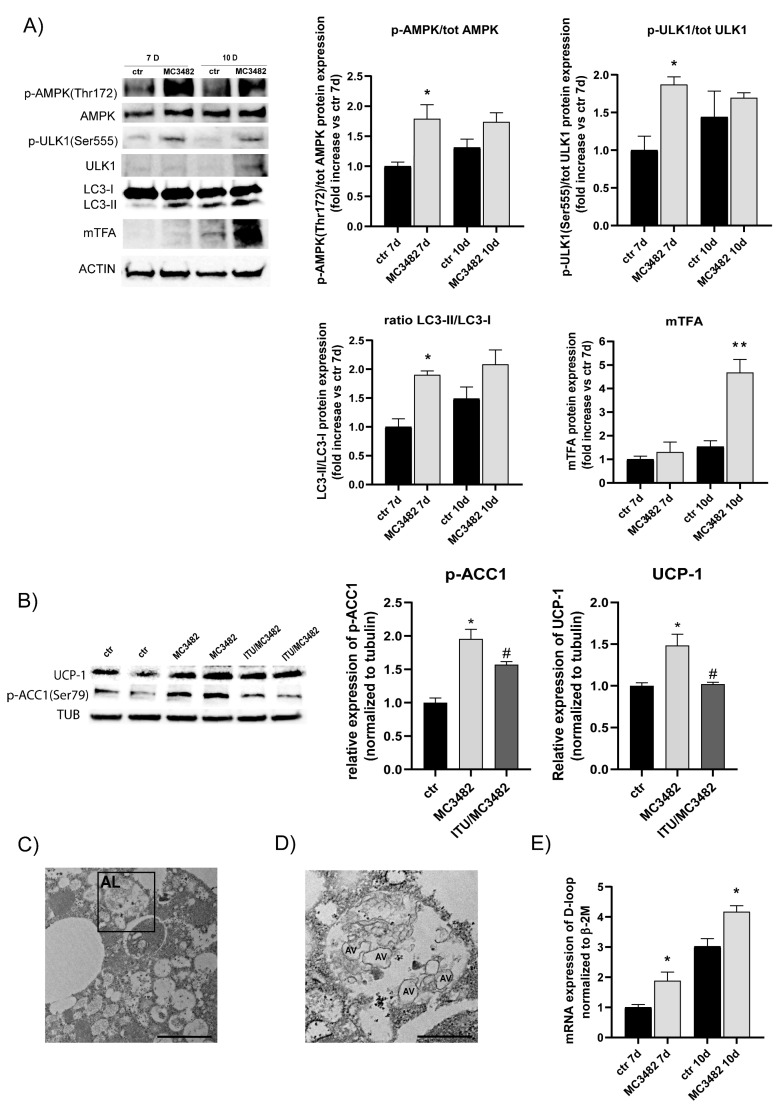
Effects of MC3482 treatment on autophagy and mitochondrial abundance. (**A**) Western blot analysis in 3T3-L1 cells treated with or without MC3482, for AMPK, authophagic and mitophagic markers, and mTFA at day 7 and 10 of differentiation. (**B**) Western blot analysis of UCP-1 and p-ACC1 at day 10 of 3T3-L1 cell differentiation upon treatment with vehicle (ctr), MC3482 or with MC3482 and the AMPK inhibitor 5-iodotubercidin (ITU). (**C**) Representative TEM images showing autophagolysosomal (AL) membranes in 3T3-L1 cells upon MC3482 treatment. Scale bar = 2 µm. (**C**) Enlargement of AL in (**D**), autophagic vacuoles are indicated as “AV”. Scale bar = 600 nm. (**E**) Relative mitochondrial DNA content measurements performed by qPCR. Values are expressed as means ± SEM of 3 independent experiments. * *p* < 0.05, ** *p* < 0.01 vs. untreated control (ctr), ^#^ *p* < 0.05 vs. MC3482, at corresponding time points.

Treatment of MC3482 led to increased activatory phosphorylation of AMPK at Thr172 in 3T3-L1 adipocytes (Figure 4A). Previous studies have shown that AMPK activity affects BAT function, regulating mitochondrial homeostasis through promotion of mitophagy [15]. In mouse iWAT, the lack of AMPK resulted in a decreased number of mitochondria, with a parallel increase in disrupted cristae, and a reduced expression of genes involved in thermogenesis [44]. On the contrary, pharmacological activation of AMPK promoted the expression of thermogenic-related genes in differentiating cultures of primary preadipocytes from mouse iWAT [44]. In accordance with these data, we observed that AMPK activation, by treatment with MC3482, was paralleled by enhancement of brown adipogenesis (Figure 2) and by an increase in the activatory phosphorylation at Ser555 of ULK1, with a concomitant rise in the protein levels of LC3-II (Figure 4A) at day 7 of differentiation, suggesting that SIRT5 inhibition was able to promote mitophagy. MC3482 treatment was able to modulate expression of BNIP3 (Figure S3), which has been shown to increase the autophagic/mitophagic flux rates [45,46]. Notably, MC3482 treatment of 3T3-L1 adipocytes resulted in increased phosphorylation of Acetyl-CoA Carboxylase 1 (ACC1) at Ser79 (Figure 4B). ACC 1 is a direct AMPK substrate which is frequently used to analyze AMPK activity [6,47], and increased levels of p-ACC1 (Ser 79) indicated AMPK activation by SIRT5 inhibition (Figure 4B). Cotreatment with MC3482 and the AMPK inhibitor 5-iodotubercidin (ITU) counteracted ACC1 phosphorylation (Figure 4B) accordingly. Cotreatment with MC3482 and ITU was also able to reduce a MC3482-driven increase in UCP-1 (Figure 4B), confirming a key role for AMPK in increasing MC3482-induced UCP-1 levels.

Of note, increased autophagy and mitophagy have been observed in SIRT5-silenced C2C12 skeletal muscle cells or in C2C12 cells treated with MC3482 in a previous study [20]. Analysis of autophagy was also performed by using transmission electron microscopy (TEM) which revealed autophagolysosomal membranes [48] moving around several autophagic vacuoles upon treatment with MC3482 (Figure 4C,D). An increase in autophagic/mitophagic flux was paralleled by higher levels of mTFA protein (Figure 4A), which were associated with a rise in mitochondrial DNA (mtDNA) in 3T3-L1 adipocytes differentiated in the presence of MC3482 (Figure 4E). These studies suggest the potential involvement of MC3482-induced autophagy in promoting mitochondrial biogenesis of adipose cells.

### 3.4. Pharmacological SIRT5 Inhibition Reduces Intracellular Lipid Storage by Promoting Lipolysis

**Figure 5:**
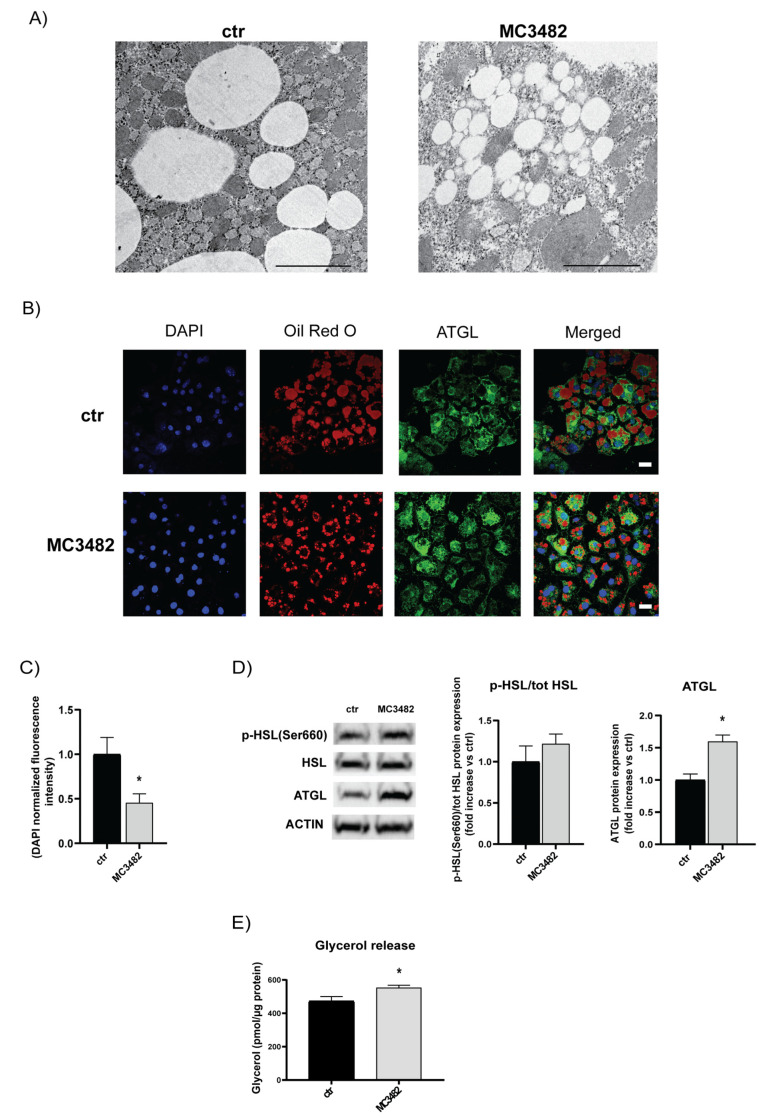
SIRT5 inhibition affects intracellular lipid droplet size and lipolysis. (**A**) Representative TEM images of intracellular lipid droplets (LDs) in 3T3-L1 adipocytes at day 10, treated or not with MC3482. Scale bars = 2 μm. (**B**) Confocal microscopy analysis of LDs (stained by using Oil Red O) and adipose triglyceride lipase (ATGL). Nuclei were stained with DAPI (blue). Scale bars = 20 μm. (**C**) Evaluation of Oil Red O staining intensity. (**D**) Western blot analysis of lipolytic proteins at day 7 of 3T3-L1 cells differentiation upon treatment with MC3482 or vehicle. (**E**) Effects of MC3482 treatment on glycerol release measured in 3T3-L1 cells culture media. Supernatants of isoproterenol-stimulated 3T3-L1 cells (day 10 of differentiation) were collected and free glycerol release was measured. Values are expressed as means ± SEM of 3 independent experiments. * *p* < 0.05 vs. untreated control cells (ctr).

In 3T3-L1 adipocytes, at day 10 of differentiation, TEM analysis showed a reduction in intracellular LD size by treatment with MC3482 (Figure 5A). Fluorescence analysis by Oil Red O staining confirmed such an effect, (Figure 5B,C) suggesting that SIRT5 inhibition could activate lipolysis. Immunofluorescence studies showed that adipose triglyceride lipase (ATGL) localized around LDs identified by using Oil Red O, suggesting a potential causal role for ATGL in promoting lipolysis (Figure 5B). Treatment with MC3482 resulted in increased levels of ATGL protein at day 7 of differentiation (Figure 5D). Analysis of hormone-sensitive lipase (HSL) showed a different expression profile, revealing that MC3482 affected neither total protein levels of HSL nor activatory phosphorylation of HSL at Ser660 (Figure 5D). Notably, increased activatory phosphorylation of ATGL at Ser406 was detected at day 10 of differentiation upon treatment with MC3482 (Figure S4), further suggesting that SIRT5 inhibition stimulates ATGL function.

In addition, lipolysis assays indicated that MC3482 treatment resulted in higher release of glycerol from 3T3-L1 adipocyte cultures stimulated with ISO (Figure 5E). Overall, these findings suggest that activatory phosphorylation of AMPK (Thr172) by MC3482 treatment (Figure 4A) may result in an increase of ATGL-mediated lipolysis which could raise intracellular levels of fatty acids to promote uncoupling respiration in brown adipocytes (see Discussion).

## 4. Discussion

Our study shows that pharmacological inhibition of SIRT5 by MC3482 treatment promotes brown adipogenesis in differentiating 3T3-L1 preadipocyte cultures. This effect was also observed in primary preadipocyte cultures derived from mouse iWAT and treated ex vivo with MC3482 (Figure 2). In fact, these primary preadipocytes are prone to differentiate into brown adipocytes [27], and such process was further stimulated by MC3482. Overall, gene and protein expression analyses (Figure 2 and Figure S1) as well as immunofluorescence, TEM and OCR studies (Figure 3 and Figure S2) showed that MC3482 treatment led to brown adipocyte differentiation. In particular, increased expression of PPAR-γ and PRDM16 (Figure S1A), two key regulators of the brown specific adipogenic program [2], and morphological changes in mitochondria and in intracellular LD size (Figure 5A,B and Figure S2) confirmed that SIRT5 inhibition was able to foster brown adipogenesis. Treatment with MC3482 promoted AMPK activation, suggesting a potential role for this kinase in stimulating brown adipogenic differentiation. As observed by Yang et al., AMPK activity is required for mouse BAT development, since AMPK ablation led to the reduced formation of BAT by suppressing the expression of *Prdm16*, which plays a key role in activating the brown adipocyte-specific gene program [49,50]. In the brown adipocyte, activity of AMPK is also required for cold-induced thermogenesis and browning of iWAT [15]. Overall, these data indicate that AMPK is critical for brown adipocyte function, and activated AMPK may account for the stimulatory effect of MC3482 on brown adipogenesis. To support this hypothesis, we observed that cotreatment of 3T3-L1 preadipocytes with MC3482 and the AMPK inhibitor ITU prevented ACC1 phosphorylation (which reflects the activation of AMPK) as well as the increase in UCP-1 levels induced by MC3482 (Figure 4B), strongly suggesting that AMPK activation plays a crucial role in mediating the effects of MC3482 on brown adipocyte differentiation.

It is possible that a causal link between SIRT5 and AMPK activity is not specific only to adipose cells, since another study by Zhang et al. has shown increased AMP/ATP ratio and enhanced activity of AMPK in cardiac tissue, further suggesting that the SIRT5 activity may have a prominent role in regulating metabolic processes modulated by AMPK [51]. In particular, AMPK has been shown to regulate mitochondrial biogenesis and mitophagy, thus enhancing cell capacity for the oxidative catabolism of both glucose and fatty acids [51]. Mitophagy consists of specific degradation of mitochondria through the autophagic flux, and transgenic mice with adipocyte-specific impairment in autophagy showed altered development of BAT with loss of mitochondria and impaired lipolysis [52]. In mouse BAT, AMPK ablation led to impaired mitophagy and altered mitochondrial function [15]. In these mice deficient for adipocyte AMPK, the analysis of mitochondrial morphology by TEM showed altered mitochondrial structure, i.e., disrupted cristae, with concomitant lower respiration rates of the mitochondria. In addition, mitophagy markers such as phospho-ULK1 Ser555 and LC3-II were decreased in BAT of these transgenic mice, suggesting that impaired mitophagy, a process which allows the clearance of damaged mitochondria and maintains mitochondrial quality, resulted in altered mitochondrial function [15,53].

Our findings show that the treatment of differentiating 3T3-L1 preadipocytes with MC3482 led to an increase in activatory phosphorylation of AMPK at Thr172, which could raise levels of phospho-ULK1 Ser555 and LC3-II (Figure 4A), thus stimulating mitophagy to maintain mitochondrial integrity and function. Interestingly, mitophagy and mitochondrial biogenesis act together to maintain mitochondrial health, which is crucial for the thermogenic activity of BAT [24]. Pharmacological AMPK activation has been shown to trigger nucleosome remodeling and reduce methylation of promoters of specific genes encoding transcription factors that stimulate mitochondrial biogenesis and function, such as PGC-1α and mTFA [54]. Notably, in 3T3-L1 adipocytes treated with MC3482, increased activity of AMPK resulted in increased levels of PGC-1α and mTFA (Figure 2B and Figure 4A), stimulating mitochondrial biogenesis, as detected by analysis of mtDNA (Figure 4E). Measurements of OCR clearly indicate that MC3482 increased maximal respiration (Figure 3B), suggesting that SIRT5 inhibition promotes mitochondrial respiration due to increased mitochondrial biogenesis. Fatty acid oxidation is required for the thermogenic activity of BAT [55]. In brown adipocytes, fatty acids are used as the energy substrate and show the ability to directly activate UCP-1. In addition, fatty acids promote the transcription activity of peroxisome proliferator-activated receptors (PPARs) that regulate the expression of genes involved in oxidative phosphorylation and thermogenesis [56]. In the adipocyte, triglyceride lipolysis to release fatty acids requires the activation of lipases such as HSL and ATGL [57]. Upon treatment of 3T3-L1 preadipocyte cultures with MC3482, we observed increased protein levels of ATGL (at day 7 of differentiation) and higher levels of activatory phosphorylation of ATGL at Ser406 (at day 10 of differentiation) (Figure S4) which enhanced its hydrolase activity [58], indicating that MC3482 promoted ATGL activation. Notably, MC3482 did not stimulate HSL function (Figure 5D), suggesting that SIRT5 inhibition specifically affects ATGL function in the adipocyte.

Increased lipolytic activity was detected in MC3482-treated 3T3-L1 adipocytes (Figure 5E) and might lead to a reduction in intracellular lipid droplet size, as observed by using TEM analysis and Oil Red O staining (Figure 5A,B). In the adipocyte, ATGL has a crucial role in conferring a brown adipocyte phenotype in mice, and adipocyte-specific ablation of ATGL converts BAT to a WAT-like tissue [58]. These transgenic mice showed impaired thermogenesis with decreased expression of UCP-1, indicating that ATGL-catalyzed lipolysis is required for maintaining UCP-1 expression and BAT phenotype. The study by Ahmadian et al. also showed that activated AMPK was able to phosphorylate ATGL at Ser406, enhancing its TAG hydrolase activity. Our findings show that MC3482 was able to specifically raise ATGL expression (Figure 5D), suggesting an increase in activity of this lipase. In addition, MC3482 also stimulated activatory phosphorylation of ATGL, potentially through the activation of AMPK (Figure 4A). Mechanistically, data by Ahmadian et al. suggest that the ATGL-dependent release of fatty acids, within the adipocyte, provides ligands for binding and activation of PPAR-α and PPAR-γ at the promoter region of UCP-1, with subsequent stimulation of UCP-1 promoter activity [58]. MC3482 might promote UCP-1 expression by activating the pathway AMPK-ATGL. Of note, another study performed in our laboratory has shown that activation of this pathway, upon mineralocorticoid receptor antagonism, increased UCP-1 protein levels in brown adipocytes [6]. A recent study has investigated the effects of SIRT5 ablation in mouse BAT, showing that SIRT5 knockout BAT displayed increased mitochondrial protein succinylation and malonylation [14]. BAT-specific SIRT5 KO mice showed impairment in mitochondrial respiration and, in particular, revealed a higher level of succinylation of UCP-1, a posttranslational modification which reduces its stability and activity [14]. Another study has shown that SIRT5 is required for brown adipogenesis through histone modification within the promoters of *Pparγ* and *Prdm16*, master regulatory genes of brown adipocyte differentiation [18]. Knockdown of SIRT5 led to repressive histone methylation at the promoter of *Pparγ* and *Prdm16* genes, resulting in reduced expression of these genes. In accordance with in vitro data, SIRT5 KO mice displayed impaired browning of iWAT in response to cold stimulus, showing lower levels of UCP-1 protein levels in this fat depot, indicating that absence of SIRT5 function led to impaired formation of brown/beige adipocyte due to defects in activation of brown adipogenesis also in vivo [18]. Our findings on the effects of MC3482 on adipocyte differentiation are in contrast with both of the above-mentioned studies [14,18]. This discrepancy might derive from the limited exposure time of the adipocyte cultures to MC3482. In our experiments, we removed MC3482 from the medium after the first 48 h of differentiation to avoid potential toxic effects. We cannot exclude that SIRT5 inhibition until terminal differentiation may lead to different effects on adipogenesis. In BAT of SIRT5 KO mice, the altered function of UCP-1, impaired mitochondria respiration and defective mitophagy [14] may derive from the permanent absence of SIRT5, which is not a comparable approach to the transient pharmacological inhibition of SIRT5 during the early stages of in vitro adipogenesis, as described in the present study. Long term treatment of differentiating preadipocytes with MC3482 might result, on brown adipocyte differentiation, in effects similar to those observed in BAT of SIRT5 KO mice [14] and in SIRT5 knockdown experiments [18]. Sirtuins regulate important cellular processes, and SIRT5 has been shown to regulate glucose, fatty acid, and nitrogen metabolism, and regulate reactive oxygen species levels [59]. Interestingly, SIRT5 involvement in regulating the growth of several types of tumors led to characterizing SIRT5 inhibitors that, however, lack selectivity for SIRT5 and are capable of inhibiting function of other sirtuins [59]. Of note, MC3482 displays no effects upon SIRT1 and SIRT3 activities, although there are no data to support the inhibition of the other sirtuins [20,59] and this might explain the activating effects of SIRT5 inhibition observed in our experiments, that are in contrast with those reported by other groups [14,18].

Altered expansion of white fat mass and impaired glucose metabolism has been associated with an impaired BAT function, suggesting that reduced activity of BAT contributes to the development of obesity [5]. In this context, pharmacological approaches aimed at stimulating BAT may enhance energy expenditure through thermogenesis and lead to weight loss and protective effects against obesity, insulin resistance and type 2 diabetes (T2D) [8]. Further preclinical studies to test MC3482 in obese mice are needed to investigate the in vivo thermogenic potential of this drug. Indeed, SIRT5 inhibition by MC3482 might offer a novel pharmacological strategy to be considered in metabolic and cardiovascular rehabilitation programs to improve quality of life of obese subjects with T2D and metabolic syndrome.

## Data Availability

Data is contained within the article or supplementary material. Supplementary materials are available at http://www.mdpi.com/xxx/s1.

## Supplementary Materials

The following are available online at https://www.mdpi.com/article/10.3390/cells10051126/s1. Table S1: sequences of primers used to perform qPCR; Figure S1: (A) Transcript analysis of PPAR-γ and PRDM16 at day 2, 7 and 10 of differentiation in 3T3-L1 preadipocytes treated with or without MC3482. (B) Transcript analysis of PGC-1α, UCP-1, CIDEA, CEBP-β, ADRB3 and DIO2 at day 10 of differentiation in 3T3-L1 preadipocytes treated with or without MC3482. Values are expressed as means ± SEM of 3 independent experiments. * *p* < 0.05, ** *p* < 0.01, *** *p* < 0.005 vs. untreated control (ctr) at corresponding time points; Figure S2: (A) TEM analysis in 3T3-L1 adipocytes (at day 10) differentiated in the presence of MC3482 which promoted physical interaction between lipid droplets (LDs) and mitochondria (M). Black arrows indicate the contact points between LD and M. Scale bars = 1 µm. (B) TEM analysis in 3T3-L1 adipocytes (at day 10) displaying the presence of mitochondria with tubular morphology in vehicle-treated cells (left side of the figure) and round-shaped mitochondria in MC3482-treated cells (right side of the figure). Scale bars = 2 µm; Figure S3: (A) Analysis of distinct components of the mitochondrial respiration in 3T3-L1 preadipocytes induced to differentiate with or without MC3482. Values are expressed as means ± SEM of 3 independent experiments. (B) Western blot analysis of BNIP3 in 3T3-L1 adipocytes treated with or without MC3482. Values are expressed as means ± SEM of 3 independent experiments; Figure S4: Western blot analysis of ATGL activation in 3T3-L1 adipocytes at day 10 of differentiation. Values are expressed as means ± SEM of 3 independent experiments. * *p* < 0.05 vs. untreated control (ctr).
