# Supplementary material for: SIRT5 Inhibition Induces Brown Fat-Like Phenotype in 3T3-L1 Preadipocytes
Source: Cells. 2021 May 7;10(5):1126. doi: 10.3390/cells10051126 (PMC8148511; doi:10.3390/cells10051126)
Supplement: Supplementary file 1 [file cells-10-01126-s001.zip › cells-1206234-supplementary.pdf]

Supplementary Materials

Table S1. sequences of primers used to perform qPCR.

	Primers, 5′-3′	
**Gene**	**Forward**	**Reverse**
β-3 adrenergic receptor(Adrb3)	TTGTCCTGGTGTGGATCGTG	TTGGAGGCAAAGGAACAGCA
β-2-microglobulin (β2M)	GTGGTGACCAAGACTCGTGA	GACAAGAACCATCCCTTCCA
Cell death-inducing DFFA-like effector A (CIDEA)	CTCGGCTGTCTCAATGTCAA	GGAACTGTCCCGTCATCTGT
Iodothyronine deiodinase 2 (DIO2)	TCCTAGATGCCTACAAACAGGTTA	CCCATCAGCGGTCTTCTCC
D-loop	GGTTCTT ACTTCAGGGCCATCA	GATTAGACCCGTTACCATCGAGAT
Peroxisome proliferator-activated receptor γ co-activator 1- α (PGC1-α)	CCGAGAATTCATGGAGCAAT	TTTCTGTGGGTTTGGTGTGA
TATA box binding protein (TBP)	GGACCAGAACAACAGCCTTC	CCGTAAGGCATCATTGGACT
Uncoupling protein 1 (UCP-1)	TCAGGATTGGCCTCTACGAC	TGCCACACCTCCAGTCATTA
Prdm16	GATGGGAGATGCTGACGGAT	TGATCTGACACATGGCGAGG
PPAR-γ	CCTCTCCGTGATGGAAGACC	CCATTGGGTCAGCTCTTGTG
Cebp-β	GCAAGAGCCGCGACAAG	GGCTCGGGCAGCTGCTT

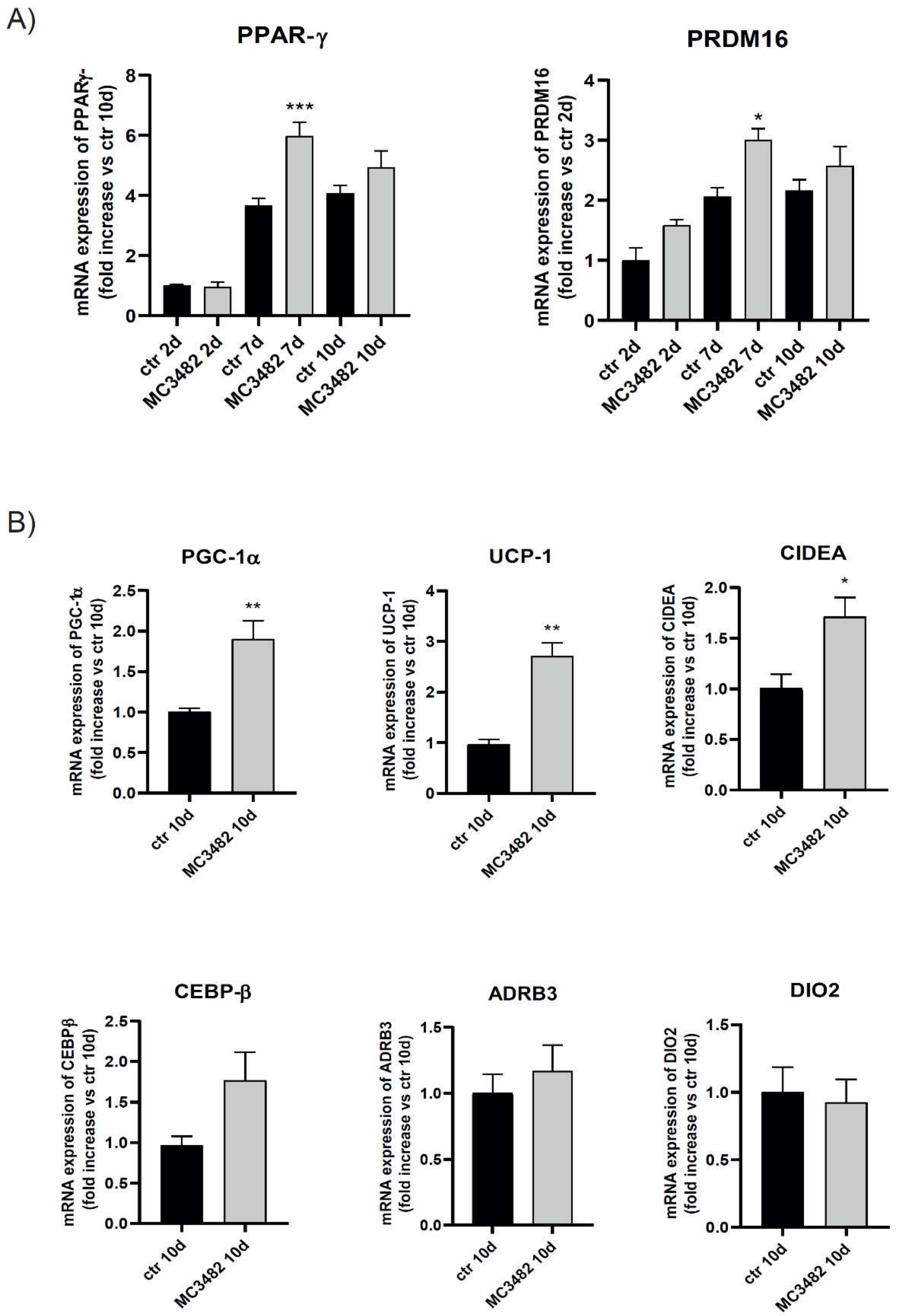

Figure S1. A) Transcript analysis of PPAR-γ and PRDM16 at day 2, 7 and 10 of differentiation in 3T3-L1 preadipocytes treated with or without MC3482. **B**) Transcript analysis of PGC1-α, UCP-1, CIDEA, CEBP-β, ADRB3 and DIO2 at day 10 of differentiation in 3T3-L1 preadipocytes treated with or without MC3482. Values are expressed as means ± sem of 3 independent experiments. * $p < 0.05$, ** $p < 0.01$, *** $p < 0.005$ *vs* untreated control (ctr) at corresponding time points.

A)

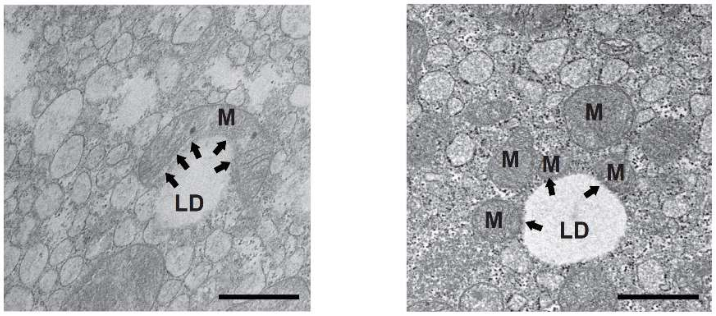

B)

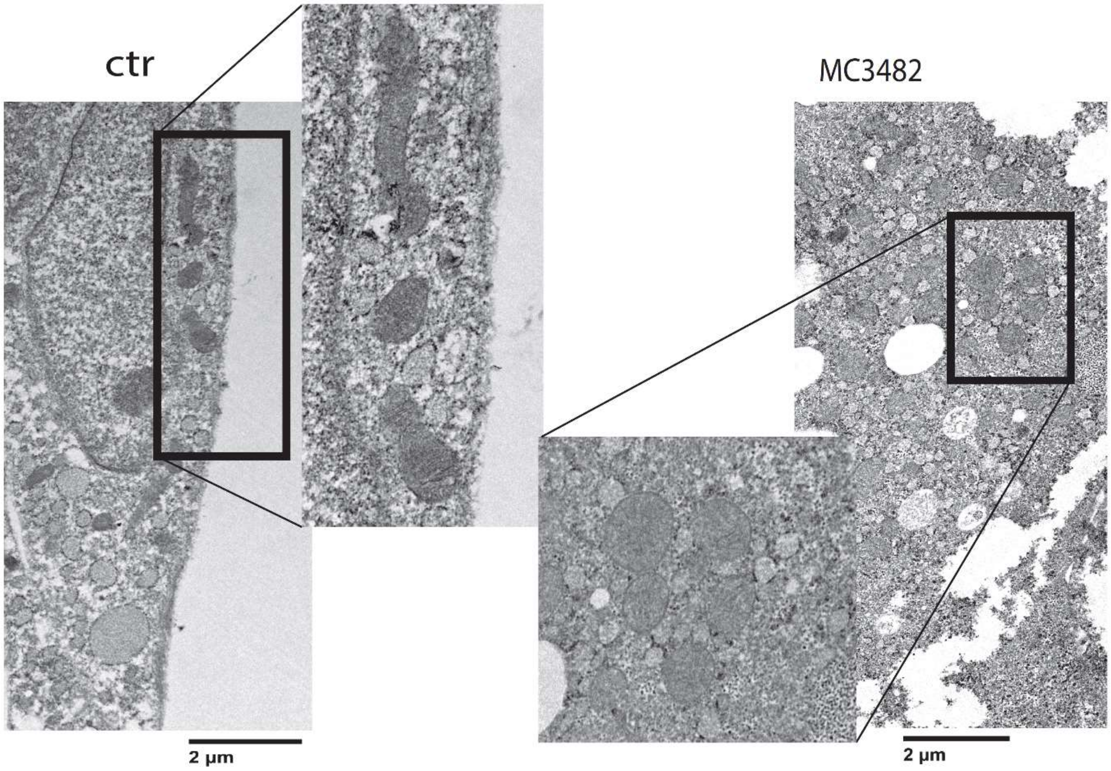

Figure S2. A) TEM analysis in 3T3-L1 adipocytes (at day 10) differentiated in the presence of MC3482 which promoted physical interaction between lipid droplets (LDs) and mitochondria (M). Black arrows indicate the contact points between LD and M. Scale bars = 1 μm. **B**) TEM analysis in 3T3-L1 adipocytes (at day 10) displaying the presence of mitochondria with tubular morphology in vehicle-treated cells (left side of the figure) and round-shaped mitochondria in MC3482-treated cells (right side of the figure). Scale bars = 2 μm.

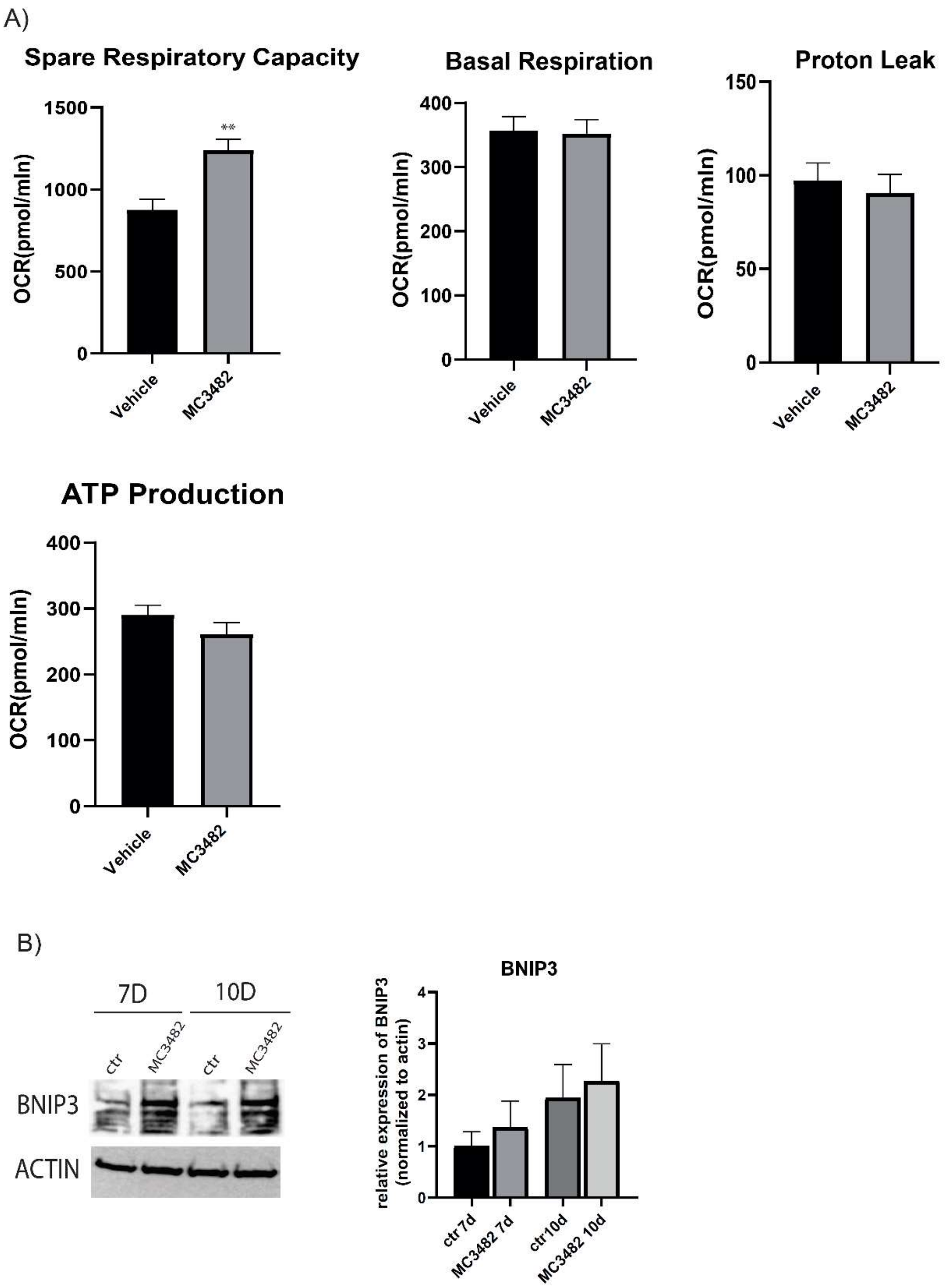

Figure S3. A) Analysis of distinct components of the mitochondrial respiration in 3T3-L1 preadipocytes induced to differentiate with or without MC3482. Values are expressed as means ± sem of 3 independent experiments. **B**) Western blot analysis of BNIP3 in 3T3-L1 adipocytes treated with or without MC3482. Values are expressed as means ± sem of 3 independent experiments.

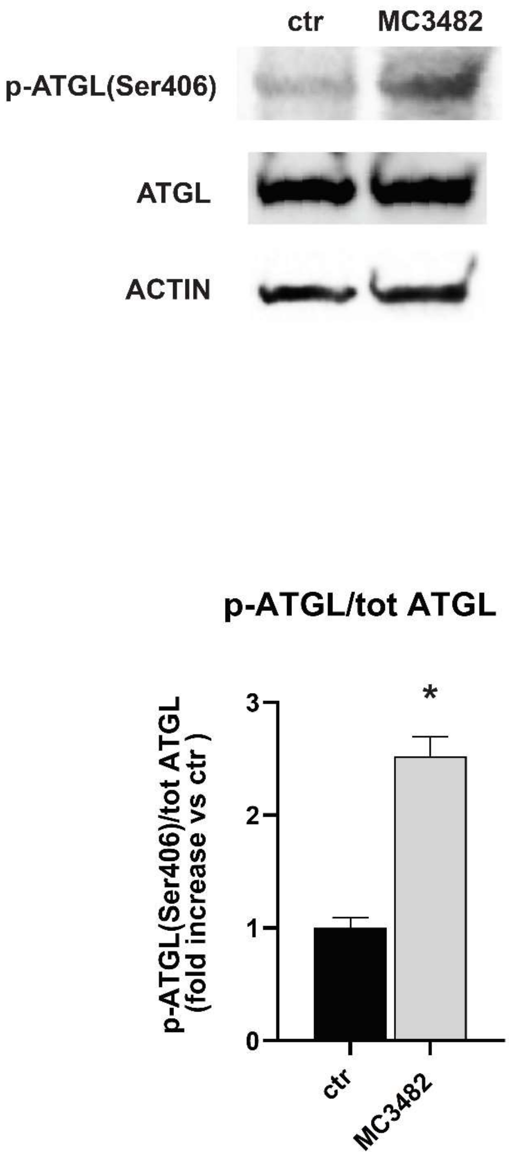

Figure S4. Western blot analysis of ATGL activation in 3T3-L1 adipocytes at day 10 of differentiation. Values are expressed as means ± sem of 3 independent experiments. * $p < 0.05$ *vs* untreated control (ctr).
